# The Effect of Maternal Obesity on Placental Autophagy in Lean Breed Sows

**DOI:** 10.3390/vetsci12020097

**Published:** 2025-01-27

**Authors:** Liang Tian, Fen Su, Xueyi Zhu, Xingyue Zou

**Affiliations:** College of Animal Science and Technology, Nanjing Agricultural University, Nanjing 210095, China

**Keywords:** pig, placenta, autophagy, obesity, fatty acid

## Abstract

This study aimed to evaluate the influence of back-fat thickness (BF), at mating of sows, on autophagy in placenta and the potential mechanism. The sows were divided into two groups according to their BF at mating: BFI (15–20 mm, *n* = 14) and BFII (21–27 mm, *n* = 14) as the maternal obesity group. The placental samples used for investigating autophagic function and fatty acid profiles were obtained by vaginal delivery. Our results demonstrated that autophagy defects were observed in placenta from BFII sows along with altered circulating and placental fatty acid profiles. Indicative of impaired autophagy, reduced autophagic vesicles as well as LC3-positive puncta were linked to decreased mRNA or protein expression of autophagy-related genes, including ATG5, ATG7, Beclin1, ATG12, LC3, LAMP1 and LAMP2 in the placenta of BFII sows (*p* < 0.05). Meanwhile, we found reduced conversion of LC3-I to LC3-II and up-regulated protein content of p62 in the placenta from BFII group (*p* < 0.05). Furthermore, excessive back-fat was also associated with increased activation of AKT/mTOR signaling and decreased mRNA content of transcription factors regulating the autophagic pathway, including PPARα and PGC1α, but increased mRNA expression of NcoR1 in placenta. Together, these findings indicate that maternal obesity incites autophagy injury in pig term placenta, which may contribute to augmented placental lipid accumulation and therefore impaired placental function.

## Simple Summary

Maternal obesity-associated excess lipid accumulation in placenta has been demonstrated to be a possible causal mechanism to provoke placental dysfunction and the subsequent adverse reproductive consequence in humans and pigs. Moreover, there is currently a growing number of studies revealing that impaired autophagy coupled with obesity induces lipid metabolic abnormality in metabolically active tissues, including muscle, liver and adipose. Despite previous studies having determined that increased back-fat thickness is associated with augmented fat deposition in placenta of sows, the effect of maternal obesity on autophagy in porcine placenta is still uncertain. In this study, animal (Landrace) was used to investigate autophagy alterations in placenta of sows with distinct back-fat thickness. We found that maternal obesity in sows during pregnancy is coupled with placental autophagy abnormalities corresponding to reduced autophagic vesicles and downregulation of the autophagy-lysosomal pathway in the placenta. Overall, these findings develop the knowledge about the influence of maternal obesity on autophagic function in porcine placenta, which may be conducive to generate some strategies in the future to promote the reproductive performance of sows.

## 1. Introduction

Emerging evidence reveals that defective autophagy was observed in human placentas from pregnancies complicated by obesity, which may contribute to the offspring programming of obesity and metabolic disease in later life, suggesting an essential role of autophagy in maintaining normal placental function and pregnancy [1,2]. As an evolutionarily conserved catabolic process that is central for cellular survival, autophagy has recently been shown to be involved in lipid catabolism and play a role in the central control of cellular lipid homeostasis [3,4]. Indeed, in vitro study has demonstrated that knockdown of an essential autophagy gene in cultured hepatocytes increased lipid droplets (LDs) in the cytoplasm due to defective catabolism [4]. Similarly, in vivo study also showed that inhibition of autophagy in mouse liver promoted massive lipid accumulation, confirming an important role of autophagy in lipolysis [5]. Accordingly, several studies have shown that energy-rich-diet-induced obesity is associated with systemic dysregulation of autophagy, suggesting a potential mechanism of lipid metabolic dysfunction in multiple highly metabolic tissues like liver, muscle and adipose [6,7].

As another metabolically active tissue, the placenta is also vulnerable to obesity-associated lipid metabolic abnormality. Recently, numerous human and animal studies have demonstrated that pregnancy complicated by maternal obesity contributes to a lipotoxic placental environment characterized by aggravated lipid deposition, which is associated with dysregulation of placental fatty acid transport and metabolism, thus resulting in adverse pregnancy results in human beings and pigs [8,9]. It is well known that non-esterified fatty acid (NEFA) plays a crucial role in fetal growth and development, especially during the late gestation [10]. Because fatty acid accumulation as triglyceride (TG) could not be transported across the placenta, its hydrolysis to NEFA is indispensable for placental fatty acid transport and therefore fetal growth [11]. In addition, excess fatty acid deposition has been demonstrated to incite placental dysfunction through increased oxidative stress, augmented inflammation and mitochondrial abnormality [12,13]. Although previous studies demonstrate the positive relationship between back-fat thickness of sows and augmented placental fatty acid deposition [9,13], the effects of maternal obesity that may induce placental dysfunction on autophagy of pig placenta still remain elusive.

Considering the regulatory role of autophagy in cellular lipid homeostasis, we tested the hypothesis that excessive back-fat of sows correlates with impaired autophagy in the pig placenta. Therefore, the aim of this research was to estimate the effects of maternal obesity in sows on placental autophagic function, and to investigate the potential proteins or pathways predicted to be involved in this process. In this study, we demonstrated that maternal obesity resulted in reduced activation of autophagy and decreased expression of autophagy-related genes in pig placenta through modifying pathways related to autophagy-lysosomal function, suggesting that maternal lipid overload may have adverse consequences on pig placental function and subsequent compromised fetal development.

## 2. Materials and Methods

### 2.1. Animal Model and Management

A total of 28 Landrace lean breed sows in the second parity were artificially inseminated with a mixture of semen samples to generate purebred litters. After weaning, the animals were divided according to back-fat thickness (BF) at mating into two groups: BFI (15–20 mm, *n* = 14) and BFII (21–27 mm, *n* = 14) as the maternal obesity group according to previous studies [14,15]. All of the experimental sows were kept in the same house with mechanically ventilated pens and compact slatted floors at the Research Facility of Nan Jing Agricultural University. Throughout the experiment, animals were fed twice daily at 09:00 h and 16:00 h with a standard grain-based diet (89.9% of dry matter, 14.5% of crude protein, 3300 Kcal/kg of metabolizable energy), and had free access to water. The ration of food offered to each sow was adjusted to days of pregnancy and calculated for fulfilling pregnancy development, consulting the nutrient requirements from NRC Nutrient Standards (2012).

### 2.2. Experimental Procedures and Sampling

All of the animals in this research were individually weighed and determined for BF at mating and at farrowing. Back-fat depth was detected using procedures as previously reported [13]. Briefly, BF at the P2 position was determined by palpation using ultrasonography (Renco). Maternal blood samples (overnight fasting) were collected in 5 mL sterile heparinized vacuum tubes (Greiner, Frickenhausen, Germany) from the sows at day 105 of pregnancy. The plasma was centrifugated at 3500× *g* for 15 min at 4 °C and stored at −20 °C for subsequent analysis. After the fetal amnion was removed, placental tissues, sampled immediately following delivery, were rinsed sufficiently in cold physiological saline solution and cut into approximately 5 cm^2^ pieces. Villous tissue was flash-frozen in liquid nitrogen and stored at −80 °C until further processing. For each pregnancy, piglets live-born and stillborn were counted and individually weighed for the analysis of reproductive performance of sows, and each placenta was also weighed for measuring placental efficiency as a ratio of litter weight to placental weight.

### 2.3. Lipid Analysis and Lipase Activity Assay

For the plasma concentrations of triglyceride (TG), total cholesterol (CHOL) and nonesterified fatty acids (NEFA) detection, a Beckman AU5811 analyzer (Beckman-Coulter, Inc., 250S.Kraemer, Boulevard Brea, CA, USA) was used to analyze maternal plasma samples at the Biochemistry Service Platform of Nan Jing Agricultural University. Commercially available porcine specific ELISA kits (RD SYSTEMS, Minneapolis, MN, USA) were used to measure plasma leptin according to the manufacturer’s instructions. The assay sensitivities were 3.2 pg/dL, 3.9 pg/dL, 0.1 pmol/L and 0.2 ng/mL for TG, CHOL, NEFA and leptin, respectively. For leptin detection, intra-assay coefficient of variation (CV) was appropriate when lower than 5%. The contents of TG, NEFA and CHOL in placental samples were determined by porcine TG, NEFA or CHOL ELISA kit, respectively (RD SYSTEMS, Minneapolis, MN, USA), following the manufacturer’s instructions. For the lipase activity measurement, tissue samples (150 mg) were homogenized in ice-cold tissue lysis buffer and the homogenate was centrifuged at 12,000× *g* for 10 min at 4 °C. Then, lipase activity measurements were performed in the supernatant via enzyme linked immunosorbent assay kits (Enzyme linked Biotechnology Co. Ltd., Shanghai, China) according to the manufacturer’s protocols.

### 2.4. Fatty Acid Composition Assessment

For quantitative analysis of fatty acid composition, fat from blood of sows or placental tissue (200 mg) was extracted with the chloroform-methanol method and transmethylated with methanolic sodium hydroxide and boron trifluoride. Nonadecanoic acid (N5252, Sigma-Aldrich, St. Louis, MO, USA) was used as an internal standard. After methylation, fatty acid composition was determined by gas chromatography (Agilent 7890A, New York, NY, USA) with standard fatty acid methyl esters (C13-CRM47885, Sigma-Aldrich, MO, USA) according to the procedure previously described [16]. Results were expressed as a percentage of total fatty acids.

### 2.5. MDC Staining and Immunofluorescence Assay

The number of autophagic vacuoles (AV) was estimated by Monodansylcadaverine (MDC) fluorescent staining as previously described [17]. Briefly, flash-frozen placental tissue sections (6 µm) from each group (10 placentas) were incubated with MDC fluorochrome (Sigma-Aldrich, St. Louis, MO, USA) for 30 min at room temperature. After washing in PBS three times, the nuclei were counterstained with 4′, 6-diamidino-2-phenylindole (DAPI) for 10 min, then, the staining was examined by inverted fluorescent microscopy. The fluorescence intensity from MDC was quantified using the Image J software (NIH Image). For LC3-positive puncta immunofluorescence analysis, placental tissue sections were cut from each sample (*n* = 10 placentas from each group) at a thickness of 7 μm by previously reported procedures [15]. Samples were then blocked in 5% BSA-supplemented PBS for 2 h at room temperature and incubated with rabbit anti-LC3 antibody (NBP1-78964, 1:200 dilution, Novus Biological, Littleton, CO, USA) overnight at 4 °C in a humidified chamber. After washing, samples were incubated for 3 h at 25 °C with goat anti-rabbit Alexa Fluor Plus 488-conjugated secondary antibody (A32731, dilution 1:1000, Thermo Fisher Scientific, Shanghai, China) and counterstained with DAPI. Confocal images were observed on confocal laser scanning microscope (Zeiss LSM 700 META, Jena, Germany) and processed with ImageJ software (NIH).

### 2.6. Transmission Electron Microscopy

For the detection of autophagic vesicle (AV) density, transmission electron microscopy (TEM) was performed as previously reported [18]. Briefly, by using 30 mg/L glutaraldehyde and 1% osmium tetroxide, placental tissue (small pieces) was fixed and embedded in Epon-812 after dehydration. The samples were then cut by an UC Enuity ultramicrotome (Leica Microsystems, WTZLAR, Germany). Ultrathin sections at a thickness of 50 nm were contrasted with 8% uranyl acetate and lead citrate after mounting on copper grids. TEM images were obtained with a Talos L120C TEM (Thermo Fisher Scientific, Waltham, MA, USA). Analysis of the TEM images was performed using the Image-ProPlus 6.0 software (Media Cybernetics, Rockville, MD, USA).

### 2.7. RNA Isolation and RT-qPCR Analysis

Total RNA was extracted from placental villous tissue from each group (14 placentas) using Trizol Reagent (Invitrogen, Carlsbad, CA, USA) and quantified by Spectrophotometer NANODROP-2000 (Thermo Fisher Scientific, Waltham, MA, USA). Five hundred nanograms of total RNA was reverse transcribed using the reverse transcription kit (TaKaRa, Tokyo, Japan). The synthesized cDNA was then used in each real-time PCR assay by the QuantStudio 7 Flex system (ABI, Foster city, CA, USA). Quantitative PCR analysis was conducted with the following program: 95 °C for 30 s; 95 °C for 5 s, 60 °C for 30 s (40 cycles); 95 °C for 15 s, 60 °C for 60 s and 95 °C for 15 s. Amplification was achieved in a 25 µL reaction system containing 2 × SYBR premix Ex Taq (TaKaRa, Tokyo, Japan) and gene-specific primers (Table S1). Primers were synthesized by Invitrogen (Shanghai, China). The reference gene GAPDH was used to normalize the expression of mRNA. The expression level of each gene was determined using the 2^−ΔΔCt^ method [19].

### 2.8. Western Blotting Analyses

Total protein from frozen placentas was extracted using RIPA buffer (KeyGen BioTECH, Nanjing, China) with a protease inhibitor cocktail (Roche Applied Science) by previously described procedures [15]. The protein concentration was measured using the Pierce BCA Protein Assay Kit (Thermo Scientific, Waltham, MA, USA). The protein extract (50 µg) was mixed with loading buffer, denatured by boiling for 5 min and separated by SDS-PAGE. After electrophoresis, proteins were transferred onto PVDF membrane (Merck Millipore, Darmstadt, Germany) and blocked in 1 × Tween-Trisbuffered saline containing 5% BSA for 60 min at room temperature. After repeated washing with the above blocking solution, the membranes were incubated with the respective antibodies. Primary antibodies against the following proteins were used: ATG7 (abs124170, 1:1000, Absin Bioscienc Inc, Shanghai, China), Beclin1 (NB110-87318, 1:1000, Novus Biological, Littleton, CO, USA), LC3B (NB100-2220, 1:1000, Novus Biological), P62/SQSTM1 (NBP1-48320, 1:2000, Novus Biological), mTOR (2972, 1:1000, Cell Signaling Technology, Danvers, MA, USA), Phospho-mTOR (5536, 1:1000, Cell Signaling Technology), AKT (9272, 1:1000, Cell Signaling Technology), Phospho-AKT (4060, 1:2000, Cell Signaling Technology) and GAPDH (2118, 1:1000, Cell Signaling Technology). After incubation with secondary antibodies, the membranes were washed and then visualized with ECL chemiluminescence reagents (KeyGen BioTECH, Nanjing, China). The blots were quantified using Amersham Image Quant 800 (Cytiva, Wilmington, DE, USA). Band densities were normalized in relevance to the GAPDH content.

### 2.9. Statistical Analysis

Experimental data from sows and placentas were analyzed as a completely randomized design by SPSS Statistics 27.0 software (IBM SPSS, Armonk, NY, USA). Each sow and her litter were considered as an experimental unit. Independent-Samples T Test was used to compare the difference between BFI and BFII groups. For analysis of the coefficient of variation (CV) for the piglets’ weights or fatty acid composition, the square root arcsine transformation for proportions was used. Results were presented as mean with SEM. Differences with a *p*-value < 0.05 or 0.01 or 0.10 were considered statistically significant, very significant and a trend, respectively.

## 3. Results

### 3.1. Characteristics of the Study Population

Back-fat depth and body weight (BW) were significantly greater at mating and at farrowing in BFII group compared to BFI group (*p* < 0.05, Table 1). The litter size, litter live size, litter weight and placental efficiency were significantly decreased in BFII sows compared to BFI group (*p* < 0.05), whereas there were no differences in placental weight between the two groups (Table 1). Consistent with increased placental efficiency, sows in BFI group had significantly greater average weight of live-born piglets than BFII sows (*p* < 0.05). BFII sows were also correlated with a greater number of low body weight (LBW) piglets (piglets with weight < 1.0 kg). Concurrently, we observed increased within-litter variation of piglet weights in BFII sows versus BFI group (*p* < 0.05).

### 3.2. Plasma Fatty Acid Profiles of the Studied Sows

The plasma parameters of the studied sows are shown in Table 2. BFII sows had significantly increased TG, NEFA and leptin levels in maternal blood compared to those in BFI group (*p* < 0.05), whereas there was no difference in plasma levels of total CHOL. Furthermore, blood fatty acid (FA) composition was measured with the use of gas chromatography (Table 2). BFII sows had 25% and 32% increases in total saturated FAs (SFAs) and palmitic acid (C16:0) (a major component of blood fat) in maternal plasma, respectively, compared to BFI group (*p* < 0.05). The proportion of stearic acid (C18:0) was also significantly increased in the blood of BFII sows compared with BFI sows (*p* < 0.05), but there was no difference in plasma level of myristic acid (C14:0) between BFI and BFII group. Of the monounsaturated fatty acids (MUFAs) analyzed, the proportions of total MUFAs and oleic acid (C18:1) were significantly higher in BFII sows than those in BFI group (*p* < 0.05). However, the palmitoleic acid (C16:1) level was not significantly different between BFI and BFII groups. In terms of polyunsaturated FAs (PUFAs), excessive back-fat decreased total PUFAs, eicosapentaenoic acid (EPA, C20:5n-3) and docosahexaenoic acid (DHA, C22:6n-3) compared to BFI sows (*p* < 0.05). In contrast, arachidonic acid (C20:4n-6) did not differ between the two BF groups, but tended to decrease in BFII sows (*p* = 0.073). These results suggest that excessive back-fat of sows was associated with dyslipidemia (systemic lipotoxicity), which is characterized by increased circulating lipid contents with higher proportions of SFAs, MUFAs as well as reduced PUFAs.

### 3.3. Altered FA Composition in the Placenta of Sows with Excessive Back-Fat

Because dyslipidemia was observed in sows with excessive back-fat, we next tested whether fatty acid profiles were changed in the placenta of BFII sows. As shown in Table 3, the placentas of BFII females had significantly higher TG, NEFA and CHOL contents than the placentas of BFI group (*p* < 0.05). Furthermore, maternal obesity did not change the proportions of total SFAs, myristic acid (C14:0) and palmitic acid (C16:0) but increased heptadecanoic acid (C17:0) and stearic acid (C18:0) (*p* < 0.05). However, placental levels of total MUFAs, palmitoleic acid (C16:1), oleic acid (C18:1) and eicosenoic acid (C20:1) were not significantly different between both groups. Furthermore, excessive back-fat of BFII sows tended to decrease linoleic acid (C18:2n-6) (*p* = 0.053), decreased α-Linolenic acid (C18:3n-3) (*p* < 0.05), and tended to decrease total PUFAs (*p* = 0.077) compared to BFI sows. In contrast, the percentages of DHA and EPA did not differ between the two BF groups.

### 3.4. Maternal Obesity Promotes Autophagy Defects in Pig Placenta

**Figure 1:**
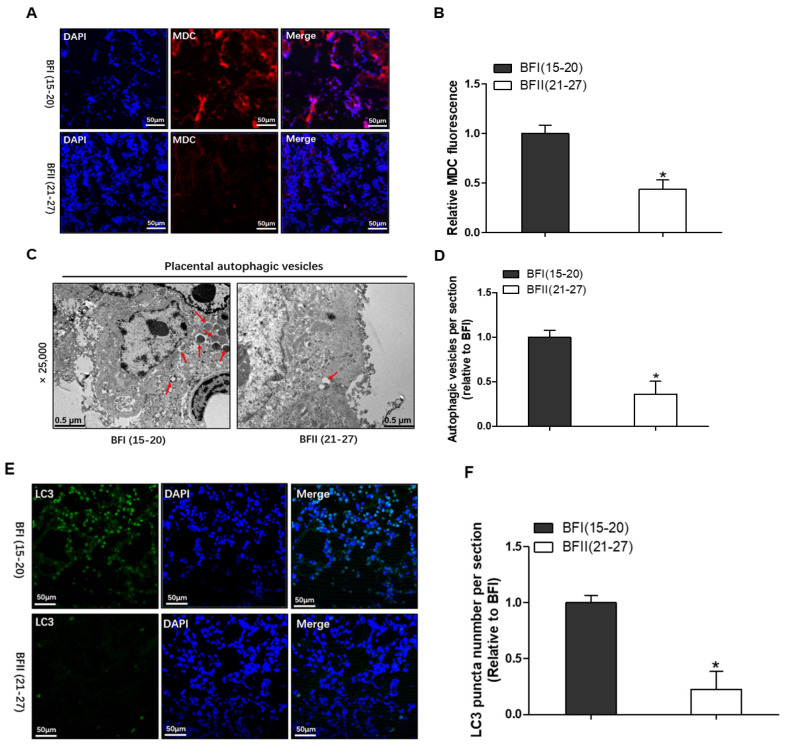
Autophagy was inhibited in placenta of sows with excessive back-fat. Monodansylcadaverine (MDC) fluorescent staining (**A**) and quantification of MDC staining (**B**) in placental tissue sections from 10 BFI and 10 BFII sows. Scale bar: 50 μm. (**C**) Autophagic vesicles were detected by transmission electron microscopy (TEM) in placentas of BFI and BFII sows. Original magnification, 25,000×. (**D**) Quantification of autophagic vesicle number per image area in whole placental tissue from 10 BFI and 10 BFII sows (analysis of 10 random images per placenta). (**E**) Placental LC3 was examined by immunofluorescence in BFI and BFII sows. Scale bar: 50 μm. Green fluorescence: LC3. (**F**) Quantification analysis of fluorescence intensity in (**E**) (*n* = 10 in each group). Results are expressed as mean ± SEM. * *p* < 0.05 compared with the BFI group; red arrow: autophagic vesicles.

There is some increasing evidence that connects dyslipidemia induced by obesity with impaired autophagy in multiple tissues, including liver and muscle [5,20]. Therefore, we next determined the impact of maternal obesity on placental autophagy. Monodansylcadaverine (MDC) fluorescent staining, which accumulates specifically in double-membrane autophagosomes, which is termed by autophagic vesicles, is widely used to assess autophagy, indicating the quantification of autophagy. Compared with the BFI group, the number of placental autophagosomes was drastically decreased in BFII sows (*p* < 0.05, Figure 1A,B). In agreement, a notably decreased number of AVs was also observed by transmission electron microscopy in the placenta from BFII group (*p* < 0.05, Figure 1C,D). LC3-positive puncta, which is a marker of the induction of autophagy, can be observed by immunofluorescence using anti-LC3 antibodies. As shown in Figure 1E, the LC3 signaling accumulated in the placenta of BFI group, while only weak signals were observed in those of BFII sows. From these findings, we surmise that excessive back-fat of sows may promote autophagy injury in placenta.

### 3.5. Effects of Maternal Obesity on mRNA and Protein Expression of Autophagy-Related Genes in Pig Full-Term Placentas

**Figure 2:**
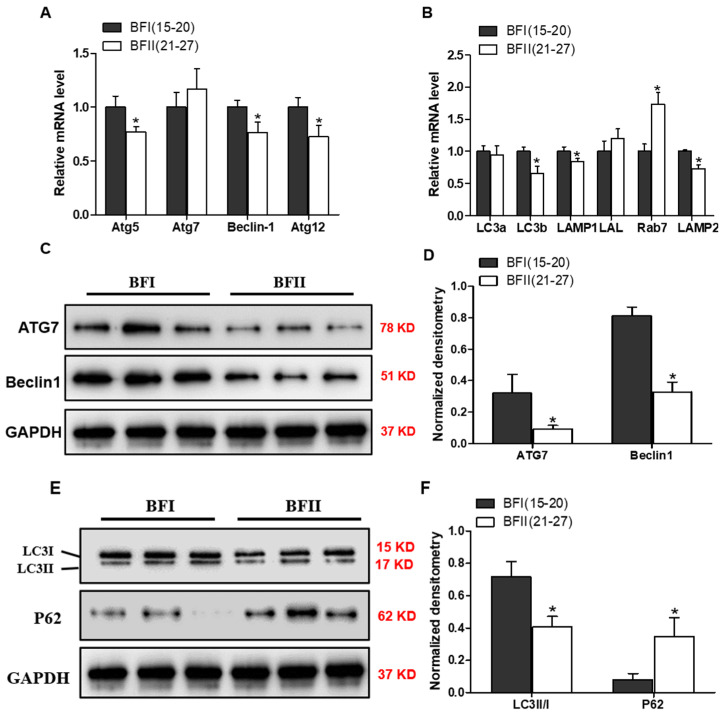
Effects of excessive back-fat on mRNA expression and protein contents of autophagy-related genes in placenta. (**A**) mRNA expression of autophagy-related genes and (**B**) autophagy-lysosomal pathway-related genes, measured by quantitative RT-qPCR, from placentas of BFI and BFII sows. (**C**,**E**) The protein expressions for LC3 and autophagosome formation were examined by Western blot in placental tissue from BFI and BFII sows. (**D**,**F**) Densitometric analysis of corresponding proteins in (**C**,**E**) by normalization to GAPDH as an internal control. The values shown represent the means ± SEM, *n* = 14 in each group. * *p* < 0.05 compared with the BFI group.

To further study the effect of excessive back-fat on placental autophagy, we then examined the mRNA and protein levels of genes involved in the autophagy-lysosomal pathway. As illustrated in Figure 2A,B, the mRNA expression of autophagy-related genes ATG5, Beclin1, ATG12 as well as autophagic marker LC3 were significantly reduced in the placenta of BFII sows compared with BFI group (*p* < 0.05), whereas there was no difference in the mRNA expression of ATG7. Moreover, the placental mRNA expression of autophagy-related genes LAMP1 and LAMP2 associated with lysosome–autophagosome fusion were decreased in BFII group compared with those in BFI sows (*p* < 0.05), but the mRNA content of Rab7, a known participant in the process of autophagosomal maturation, was higher in BFII group (*p* < 0.05) than BFI group, without a significant change in lysosomal acid lipase LAL (Figure 2B).

Additionally, we examined protein expressions of autophagy using immunoblot. Compared with the BFI group, protein expression levels of ATG7 and Beclin1 involved in autophagosome formation were obviously lower in BFII sows (*p* < 0.05, Figure 2C,D). Furthermore, a reliable marker of autophagy is the conversion of the ATG8 (LC3) protein from LC3-I to LC3-II by conjugation with phosphatidylethanolamine located in the autophagosome membrane. Thus, this conversion could reflect the autophagosome formation within a cell [21]. Western blot analysis showed that excessive back-fat dramatically reduced the conversion of LC3-I to LC3-II in the placenta of BFII sows (*p* < 0.05, Figure 2E). In contrast, the protein content of p62, another widely used marker of autophagy defects [22], was increased in placenta from BFII group compared with BFI sows (*p* < 0.05, Figure 2E,F). These data indicated that autophagy was significantly downregulated in the placenta of BFII sows with excessive back-fat.

### 3.6. Effects of Maternal Obesity on mRNA Expression of Lipolysis-Related Genes, the Activity of Lipolytic Lipase in Pig Full-Term Placentas

**Figure 3:**
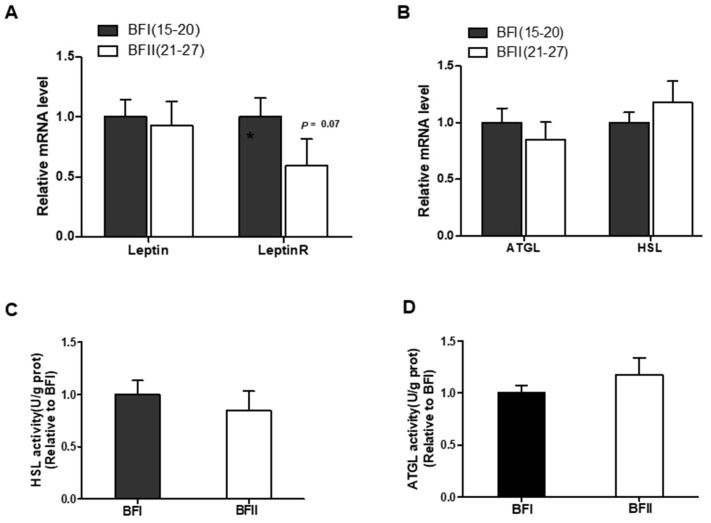
The effects of excessive back-fat on lipolysis-related genes as well as the activity of lipolytic lipase in placenta. (**A**,**B**) mRNA expressions of lipolysis-related genes in placenta of BFI and BFII sows. (**C**,**D**) Placental lipase HSL (**C**) and ATGL (**D**) activity were evaluated in BFI and BFII sows. Results are shown as mean ± SEM.

Having determined that impaired autophagy was coupled with increased lipid accumulation in placenta of BFII sows, we subsequently asked whether higher placental lipid content in BFII sows is attributed to decreased lipolysis mediated solely by the actions of compromised autophagy. Hence, we next tested whether the mRNA abundance of lipolysis-related genes or activity of lipolytic lipase was changed in placentas of BFII sows. As shown in Figure 3A, placental leptin receptor (Leptin R) mRNA expression showed a decreased tendency (*p* = 0.07) in BFII sows, despite leptin mRNA expression not changing. However, mRNA expression of ATGL and HSL (two key lipolysis-related genes), as well as the activity of lipolytic lipases in placenta, showed no differences between the BFI and BFII sows (Figure 3B–D).

### 3.7. Effects of Maternal Obesity on Key Molecules Regulating Placental Autophagic Function

**Figure 4:**
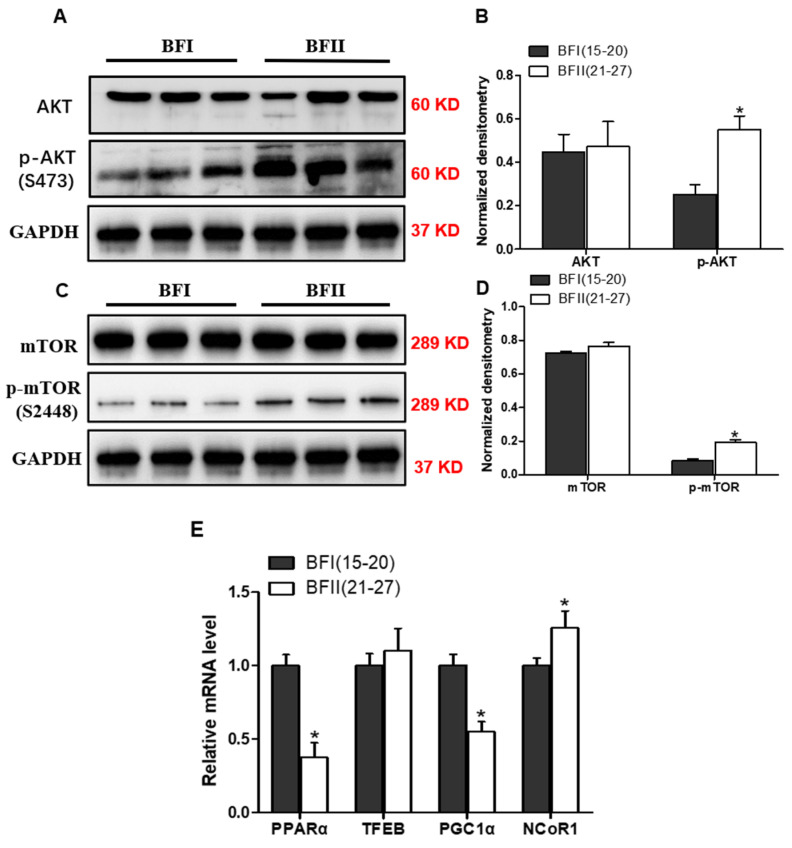
The effect of excessive back-fat on regulators of autophagy in placenta. (**A**,**C**) Western blot analysis of key molecules regulating autophagy (AKT, phos-AKT, mTOR and phos-mTOR) in placentas of BFI and BFII sows. (**B**,**D**) Densitometric analysis of corresponding proteins in (**A**,**C**) by normalization to GAPDH as an internal control. (**E**) mRNA levels of transcription factors involved in the regulation of autophagy, including PPARα, TFEB, PGC1α and NcoR1. Values are expressed as mean ± SEM. * *p* < 0.05 compared with the BFI group. *n* = 14/group.

In order to further investigate the mechanisms of impaired autophagy in placenta from BFII sows, immunoblot and quantitative PCR analysis of key regulators of autophagy were performed. As shown in Figure 4, the protein expression of phos-AKT (S473) and phos-mTOR (S2448) were increased (*p* < 0.05) in the placenta of BFII sows versus placenta from BFI sows (Figure 4A,C). Concerning transcriptional regulation of autophagy, we investigated the mRNA content of transcription factors involved in the regulation of the autophagy-lysosomal degradative pathway, including PPARα, TFEB, PGC1α and NcoR1 [23]. As illustrated in Figure 4E, excessive back-fat of BFII sows incited a significant decrease (*p* < 0.05) in mRNA levels of PPARα and its coactivator PGC1α (up-regulating autophagy-lysosomal function), whereas elevated mRNA expression of NcoR1 (a transcriptional corepressor associated with PPARα) was observed in the placenta from BFII sows (*p* < 0.05). Moreover, the mRNA level of TFEB, a key regulator of autophagy-lysosomal gene transcription, showed no difference between the two BF groups.

## 4. Discussion

**Figure 5:**
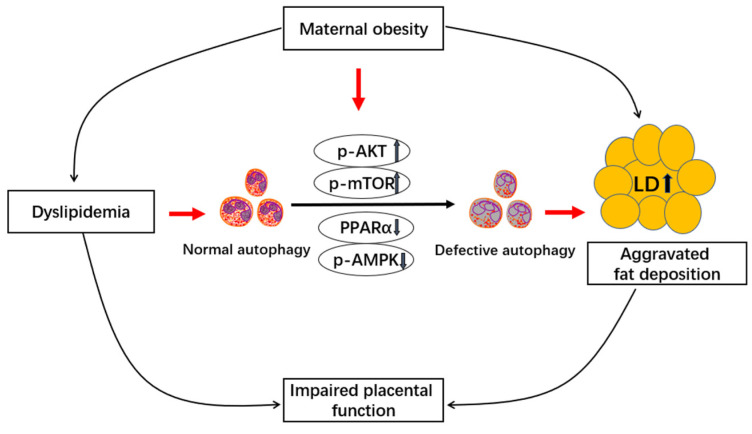
A hypothetical model of the impact of maternal obesity on autophagy in the full-term porcine placenta. Excessive back-fat aggravates dyslipidemia (systemic lipotoxicity), which may induce autophagy defects and therefore increased placental lipid accumulation. Maternal obesity promotes autophagy injury by activating the AKT/mTOR pathway and reducing AMPK and PPARα activation. ↑, Up-regulation of gene expression or increased concentration of metabolite. ↓, Downregulation of gene expression or decreased concentration of metabolite. Arrows indicates a positive regulation. LD, lipid droplet; P, phosphorylation.

As a highly metabolically active organ, the placenta plays a pivotal role in maintaining the normal growth and development of fetus. Emerging studies revealed that autophagy is essential for maintaining normal placental function and pregnancy [2]. Accordingly, dysregulation of autophagy is observed in the placenta of human beings associated with obese pregnancy, suggesting a role for defective autophagy in lipid overload-induced placental dysfunction [1]. Moreover, autophagy has recently been shown to be involved in maintaining cellular lipid homeostasis in multiple tissues such as liver and adipose [4,24]. Despite several studies demonstrated that maternal obesity promotes aggravated fat deposition in placenta, resulting in placental dysfunction and subsequent adverse pregnancy outcomes in pigs [9,13], there is a paucity of data on the effect of excessive back-fat of sows during pregnancy on autophagy in pig placenta. Several novel findings are obtained from the current research. First, we identified that impaired autophagy was observed in term placenta from BFII sows along with dyslipidemia and altered placental fatty acid profiles. Second, we demonstrated that increased lipid accumulation in the placenta of BFII sows was correlated with decreased lipolysis mediated by the actions of autophagy defects rather than cytosolic lipases. Third, our data identified a distinct downregulation of key genes involved in autophagy-lysosomal pathway and activation of AKT/mTOR pathway and inhibition of PPARα signaling, providing the potential mechanisms through which maternal obesity incites pig placental autophagy injury. Together, these data suggest that maternal obesity aggravates a lipotoxic placental environment conducive to impaired autophagy (summarized in Figure 5), which may adversely impact porcine placental function and fetal development.

In the present study, BFII sows with excessive back-fat had the trend for a higher adiposity compared with BFI sows, as evidenced by the analysis of maternal phenotypes and blood metabolic parameters, despite the fact that the sows from both groups had the same feeding management. Several studies have addressed that placenta afflicted by maternal obesity is exposed to a detrimental intrauterine milieu characterized by increased circulating inflammatory cytokines and fatty acids (lipotoxic insults), resulting in placental lipotoxicity and detrimental consequences on fetal development [25,26]. Accordingly, dyslipidemia observed in BFII sows was associated with lower birth weight of live-born piglets, increased number of LBW piglets and greater within-litter variation of piglet weights, which indicates the presence of placental abnormality. In this trial, we observed higher lipid contents but decreased proportion of α-Linolenic acid (C18:3n-3) along with a trend for fewer percentages of total PUFAs and linoleic acid (C18:2n-6) from the placentas of BFII sows, which may be indicative of the presence of reduced placental PUFA uptake. Of note, it has been demonstrated that placental tissue levels of PUFA are positively correlated with its levels in maternal plasma during late gestation [27]. Consistently, a reduction in the proportions of circulating total PUFAs, EPA and DHA was observed in BFII sows. Taken together with the observation that decreases in placental unsaturated fatty acid (UFA) uptake reported in pregnancy obesity may drive lipid storage in placenta, which can ultimately cause a lipid deficit to the fetus [28,29], we surmise that dyslipidemia in embryonic life due to maternal obesity could compromise fatty acid uptake priority and composition in pig placenta, resulting in abnormality of placental fatty acid metabolism and transport and therefore an adverse consequence for the reproductive performance of pigs.

There is currently emerging evidence showing that saturated fatty acids impair the placental trophoblast invasion and deteriorate autophagy in human extravillous trophoblasts, and these effects are reversed by UFA treatment, suggesting that the dysregulated serum free fatty acids due to maternal obesity could be a major cause of impaired autophagic function of placental trophoblasts [30]. Consistent with this notion, we found that there was a significant decrease in the numbers of LC3-positive puncta and autophagic vesicles (quantitative indexes of autophagosomal formation) in the placenta of BFII sows versus BFI group, in agreement with previous studies [31]. These findings were further confirmed by a decrease in the expression levels of autophagy-related genes (ATG5, ATG7, Beclin1, ATG12, LC3, LAMP1 and LAMP2) and the conversion of LC3-I to LC3-II, and a concomitant increase in expression of p62/SQSTM1 in the placenta of BFII sows, which suggested that dyslipidemia due to maternal obesity may contribute to autophagy injury in pig placenta. However, a complete understanding of the precise molecular mechanisms by which dyslipidemia impairs autophagy in porcine placenta associated with excessive back-fat needs to be further investigated.

There exists a substantial amount of data demonstrating that the lysosome-mediated catabolic process, called autophagy, is involved in LDs lipolysis, suggesting that the breakdown of LD-stored TG is not attributed solely to the actions of cytosolic hydrolytic enzymes or lipases [4,32]. Thus, to further confirm the contribution of impaired autophagy to the increased lipid accumulation in placenta of BFII sows, we next investigated whether the actions of cytosolic lipases are involved in this process. Consistent with our expectation, we found that there was no difference in the mRNA expression and the activity of lipolysis enzymes ATGL and HSL between BFI and BFII sows, which is probably attributed to unaltered mRNA expression of Leptin and Leptin R in placenta of BFII sows compared with BFI sows. Leptin signaling has also been shown to stimulate lipolysis in adipose tissue by up-regulating HSL and ATGL expressions and activities [33]. Considering that obesity-associated lipid accumulation in placenta may be caused by changes in placental lipid handling, such as impaired lipid transfer, decreased lipolysis or FA oxidation (FAO) and increased esterification [34], these above results suggested that defective autophagy occurring in the placentas of sows with excessive back-fat might represent one of the mechanisms contributing to increased placental lipid accumulation.

To further explore the mechanisms that adversely regulate autophagy in placenta from BFII sows, we initially investigated the core gene expression of the AKT/mTOR pathway by Western blot analysis. As a member of key nutrient-sensitive kinases, mTOR has been intensively investigated due to its pivotal role in regulating energy homeostasis and nutrient sensing of the autophagy pathway [35]. Indeed, the AKT/mTOR pathway is one of most critical molecules that inhibits autophagy in response to metabolic stress [36]. Consistent with our expectation, the immunoblot measurement confirmed that there was increased activation of AKT and mTOR in placenta from BFII sows, suggesting that activation of AKT/mTOR signaling by lipid overload could be responsible for autophagy injury in the placenta of sows with excessive back-fat. Moreover, AMPK has also been shown to be crucial in the induction of autophagy via mTOR inhibition in response to energy depletion [35]. Consistently, our previous studies revealed that maternal obesity is associated with decreased activity and expression of AMPK in porcine placenta [15], suggesting that AMPK may be involved in regulation of autophagy abnormality in placenta of BFII sows. Of note, the regulation of autophagy has also been shown to be mediated by multiple transcription factors, including PPARα, TFEB, PGC1α and NcoR1 [37]. Several studies have linked dysregulation of autophagy-related transcription factors to abnormal placental function and adverse pregnancy outcomes [38]. In agreement, downregulated expression of PPARα and PGC1α and up-regulated mRNA content of NcoR1 were observed in the placenta from BFII sows compared with BFI group. However, the precise molecular mechanisms by which these regulators manipulate autophagy in placenta of sows with excessive back-fat need to be further investigated.

## 5. Conclusions

In conclusion, our data show that maternal obesity-induced dyslipidemia is associated with changes in fatty acid profiles and downregulation of autophagy in the pig placenta. Moreover, our findings identify that augmented placental lipid accretion may be attributed to impaired autophagy, but not actions of cytosolic lipolysis enzymes in placenta of sows with excessive back-fat, which may be mediated through the activation of the AKT/mTOR pathway and inhibition of AMPK and PPARα signaling. Thus, our findings suggest that maternal obesity is associated with autophagy injury in porcine placenta, which could be considered as a potential mechanism to adversely affect placental lipid homeostasis, and therefore impair pig placental function and fetal growth.

## Figures and Tables

**Table 1:** Maternal and neonatal characteristics of sows studied ^†^.

Parameter	BFI (15–20 mm)	BFII (21–27 mm)	SEM	*p*-Value
a. Maternal characteristics				
BF at mating, mm	17.13 ^a^	24.31 ^b^	0.24	0.031
BW at mating, kg	170.58 ^a^	175.89 ^b^	0.31	0.042
BF at farrowing, mm	18.42 ^a^	26.51 ^b^	0.22	0.019
BW at farrowing, kg	216.32 ^a^	223.82 ^b^	0.49	0.036
b. Offspring measures				
Litter size ^‡^	15.15 ^a^	12.89 ^b^	0.61	0.037
Number of piglets born alive	13.01 ^a^	11.29 ^b^	0.51	0.028
Litter weight, kg	20.34 ^a^	17.56 ^b^	0.41	0.021
Birth weight ^§^, kg	1.49 ^a^	1.33 ^b^	0.27	0.033
Piglets with low birth weight ^¶^, n	0.95 ^a^	1.42 ^b^	0.17	0.012
CV for birth weight, % ^⁋^	20.35 ^a^	24.45 ^b^	1.73	0.017
Placental weight, kg	3.30	3.43	0.23	0.322
Placental efficiency ※	6.12 ^a^	5.21 ^b^	0.18	0.02

BF, Back-fat thickness; BW, body weight; SEM, standard error of the means. ^†^: Results are expressed as means with SEM for BFI (*n* = 14) and BFII (*n* = 14). Means within the same row with different superscripts mean significant difference (*p* < 0.05). ^‡^: The number of live-born and stillborn piglets in litter. ^§^: Birth weight of born alive piglets. ^¶^: Piglets with weight < 1.0 kg. ^⁋^: The coefficient of variation (CV) for the piglets’ weights was calculated by dividing the standard deviation (SD) of each live-born piglet’s weight within the litter by the average of those values, expressing it as a percentage. ^※^: A ratio of litter weight to placental weight.

**Table 2:** Blood fatty acid profiles of the studied sows ^†^.

Parameter	BFI (15–20 mm)	BFII (21–27 mm)	SEM	*p*-Value
TG, mg/dL	22.51 ^a^	34.38 ^b^	2.16	0.016
NEFA, mmol/L	0.25 ^a^	0.40 ^b^	0.02	0.021
CHOL, mg/dL	34.72	36.33	4.19	0.301
Leptin, ng/mL	13.45 ^a^	19.22 ^b^	1.98	0.029
SFA ^‡^, %				
C14:0 (Myristic)	0.62	0.86	0.11	0.323
C16:0 (Palmitate)	17.86 ^a^	23.73 ^b^	1.14	0.034
C18:0 (Stearic)	13.51 ^a^	16.13 ^b^	0.71	0.042
Total SFA	35.65 ^a^	44.72 ^b^	5.13	0.031
MUFA ^‡^, %				
C16:1 (Palmitoleic)	0.95	1.16	0.21	0.51
C18:1 (Oleic)	23.24 ^a^	28.33 ^b^	2.41	0.024
Total MUFA	27.41 ^a^	33.21 ^b^	3.13	0.011
PUFA ^‡^, %				
C20:4n-6 (Arachidonic)	6.32	4.9	0.65	0.073
C20:5n-3 (Eicosapentaenoic)	0.06 ^a^	0.02 ^b^	0.006	0.014
C22:6n-3 (Docosahexaenoic)	0.41 ^a^	0.23 ^b^	0.26	0.033
Total PUFA	36.60 ^a^	21.93 ^b^	3.91	0.008

BF, Back-fat depth; TG, triglyceride; NEFA, non-esterified fatty acid; CHOL, total cholesterol; SFA, saturated fatty acid; MUFA, monounsaturated fatty acid; PUFA, polyunsaturated fatty acid; SEM, standard error of the means. ^†^: Results are expressed as means with SEM for BFI (*n* = 14) and BFII (*n* = 14). Means within the same row with different superscripts mean significant difference (*p* < 0.05); ^‡^: The fatty acid composition (% of total fatty acids) in plasma of the studied sows.

**Table 3:** Placental fatty acid composition of the studied sows ^†^.

Parameter	BFI (15–20 mm)	BFII (21–27 mm)	SEM	*p*-Value
TG, mg/g	48.73 ^a^	67.55 ^b^	6.35	0.033
NEFA, mg/g	0.22 ^a^	0.38 ^b^	0.03	0.023
CHOL, mg/g	28.55 ^a^	38.75 ^b^	2.62	0.041
SFA ^‡^, %				
C14:0 (Myristic)	1.92	1.67	0.27	0.237
C16:0 (Palmitate)	26.88	27.53	1.54	0.432
C17:0 (Heptadecanoic)	1.53 ^a^	1.89 ^b^	0.11	0.034
C18:0 (Stearic)	18.22 ^a^	20.73 ^b^	1.16	0.045
Total SFA	46.77	49.11	5.43	0.372
MUFA ^‡^, %				
C16:1 (Palmitoleic)	1.54	1.66	0.24	0.233
C18:1 (Oleic)	39.33	41.22	2.76	0.763
C20:1 (Eicosenoic)	0.15	0.18	0.38	0.375
Total MUFA	42.18	43.73	3.34	0.274
PUFA ^‡^, %				
C18:2n-6 (Linoleic)	7.21	4.71	0.47	0.053
C18:3n-3 (α-Linolenic)	1.62 ^a^	0.67 ^b^	0.15	0.044
C20:5n-3 (Eicosapentaenoic)	0.19	0.21	0.05	0.541
C22:6n-3 (Docosahexaenoic)	0.051	0.048	0.006	0.367
Total PUFA	10.61	6.93	1.69	0.077

BF, Back-fat thickness; TG, triglyceride; NEFA, non-esterified fatty acid; CHOL, total cholesterol; SFA, saturated fatty acid; MUFA, monounsaturated fatty acid; PUFA, polyunsaturated fatty acid. SEM, standard error of the means. ^†^: Results are expressed as means with SEM for BFI (*n* = 14) and BFII (*n* = 14). Means within the same row with different superscripts mean significant difference (*p* < 0.05). ^‡^: The fatty acid composition (% of total fatty acids) in placenta of the studied sows.

## Data Availability

No original data were stored in a public repository. The data that support the study findings are available upon request.

## Supplementary Materials

The following supporting information can be downloaded at: https://www.mdpi.com/article/10.3390/vetsci12020097/s1, Table S1: Primer sets used for real-time PCR.
