# Supplementary material for: The Effect of Maternal Obesity on Placental Autophagy in Lean Breed Sows
Source: Vet Sci. 2025 Jan 27;12(2):97. doi: 10.3390/vetsci12020097 (PMC11861729; doi:10.3390/vetsci12020097)
Supplement: Supplementary file 1 [file vetsci-12-00097-s001.zip › vetsci-3337115-supplementary.pdf]

Table S1 Primer sets used for real-time PCR

Gene name[1)]	Productsize (bp)	Direction	Sequence, 5'-/- 3'
PPARG	184	Forward:	GATTTCTCCAGCATTTCCA
		Reverse:	GCTCTTCGTGAGGTTTGTT
C/EBPα	108	Forward:	GGTGGACAAGAACAGCAACG
		Reverse:	AGGCACCGGAATCTCCTAGT
SREBP-1c	218	Forward:	GCGACGGTGCCTCTGGTAGT
		Reverse:	CGCAAGACGGCGGATTTA
FAS	98	Forward:	CACTCCAAGCAGGCGAACAC
		Reverse:	CGAAGGGAAGCAGGGTTGAT
ACCα	129	Forward:	ATGTTTCGGCAGTCCCTGAT
		Reverse:	TGTGGACCAGCTGACCTTGA
SCD1	137	Forward:	TGGGCGTTTGCCTACTATCT
		Reverse:	TTCTGGAATGCCATCGTGTT
ACSL1	126	Forward:	ACCAGACCAACCCTATGAATG
		Reverse:	GAGCGAAGATGCCGACGAAA
Leptin	113	Forward:	CCCTCATCAAGACGATTGTCA
		Reverse:	GGTTCTCCAGGTCATTCGATA
Leptin R	107	Forward:	CCCTCATCAAGACGATTGTCA
		Reverse:	GGTTCTCCAGGTCATTCGATA
ATGL	143	Forward:	ACCTGTCCAACCTGCTGC
		Reverse:	GCCTGTCTGCTCCTTTATCCA
HSL	89	Forward:	ACCCTCGGCTGTCAACTTCTT
		Reverse:	TCCTCCTTGGTGCTAATCTCGT
ATG5	122	Forward:	CCATCAATCGGAAACTCA
		Reverse:	AGCCACAGGACGAAAGAG
ATG7	137	Forward:	ATTGCCTGGTGGGTGGTA
		Reverse:	GGTGATGCTGGAGGAGTT
Beclin1	104	Forward:	CGGCTCCTATTCCATCAA
		Reverse:	AGGATACCCAAGCAAGAC
ATG12	91	Forward:	CCTTCTTCCGCTTCAGTT
		Reverse:	TGTCTCCCACAGCCTTTA
L3A	126	Forward:	CCTCAGACCGGCCTTTCA
		Reverse:	GCTGCTTCTCACCCTTGTAG
L3B	135	Forward:	AGCCTTCTTCCTGTTAGTG
		Reverse:	TTCATTCCGAAAGTCTCC
LAMP1	80	Forward:	CTCTAAGGGCACCAAGACGG
		Reverse:	TGTTGCTCACGCACCTGTAC
LAMP2	82	Forward:	TCTGAAGGAGGTGAATGT
		Reverse:	ATCCCAGTAGCTGAGATT
LAL	98	Forward:	GTGGGTCATTCTCAAGGT
		Reverse:	ACAGGAGCCAAGGCAAAA
Rab7	150	Forward:	AAGCGACAATAGGAGCAG
		Reverse:	ACACCAGAACGCAGCAGT

Gene name[†]	Productsize (bp)	Direction	Sequence, 5’-/- 3’
PPARα	120	Forward:	CTGGACGACAGTGACCTTT
		Reverse:	TTTGAGCACATGCACGATA
TFEB	109	Forward:	AACAGTGCTCCCAACAGCC
		Reverse:	TGAAGCCCAGGACATCGTC
PGC1α	122	Forward:	ACAATGAGCCTTCAAACA
		Reverse:	ATCAAATGAGGGCAATCC
NCoR1	105	Forward:	GGAGAAGTGGATACGAGCAGT
		Reverse:	GGAAGTGGGAGTCGGAAA
GAPDH	233	Forward:	CCACAACATACGTAGCACCACGATC
		Reverse:	CCTTCATTGACCTCCACTACATGGT

[1)] All porcine gene sequences.
