# Supplementary figures and images for: The Effect of Maternal Obesity on Placental Autophagy in Lean Breed Sows
Source: Vet Sci. 2025 Jan 27;12(2):97. doi: 10.3390/vetsci12020097 (PMC11861729; doi:10.3390/vetsci12020097)

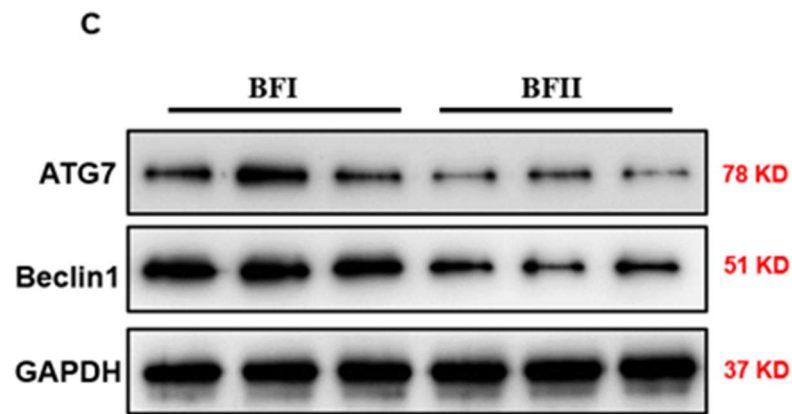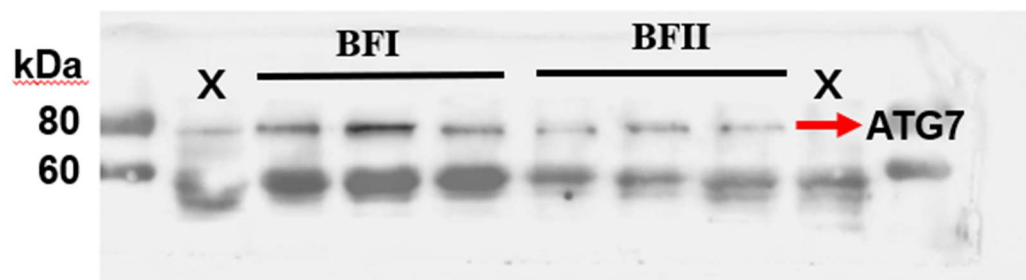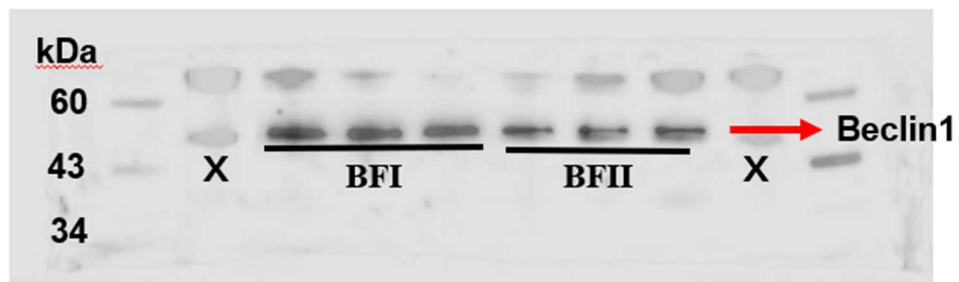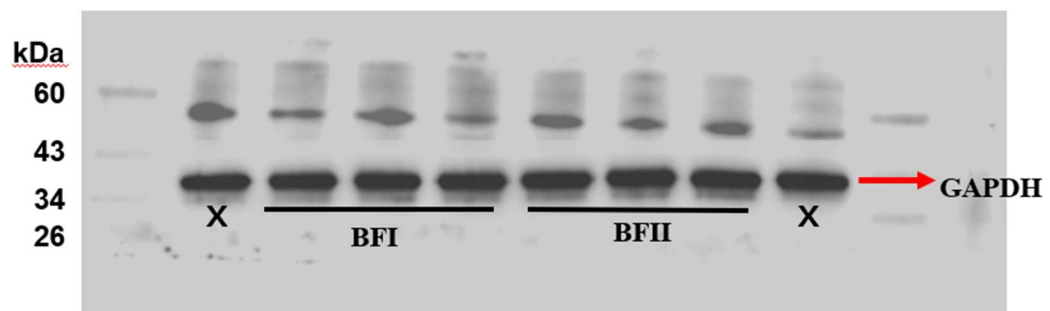

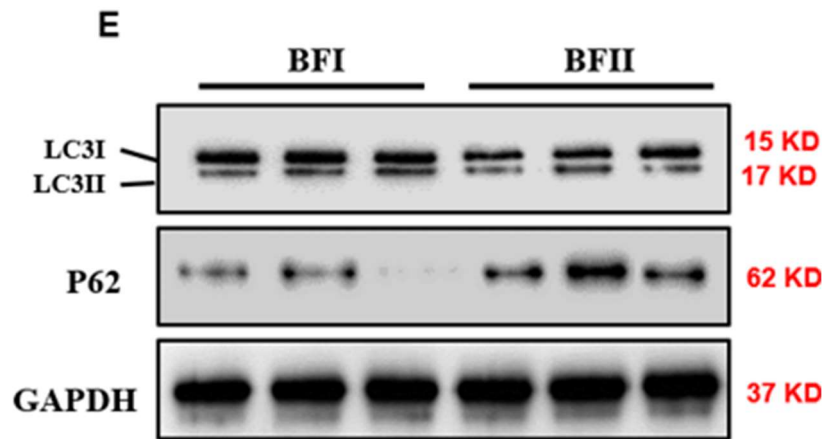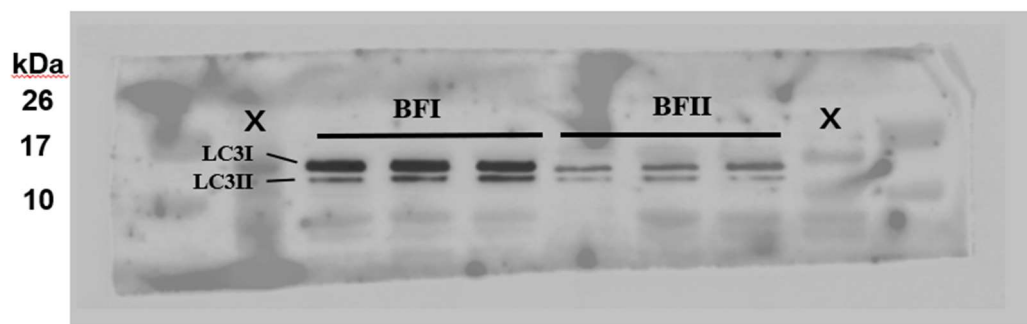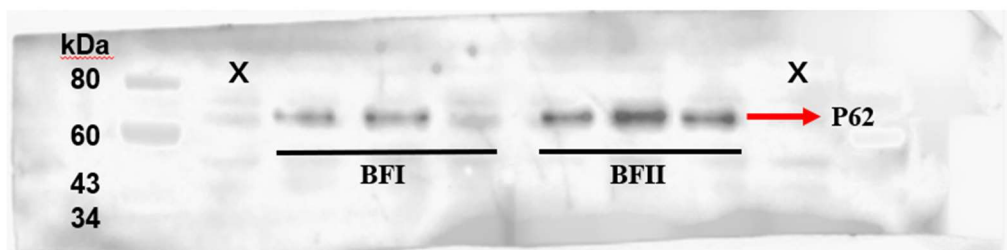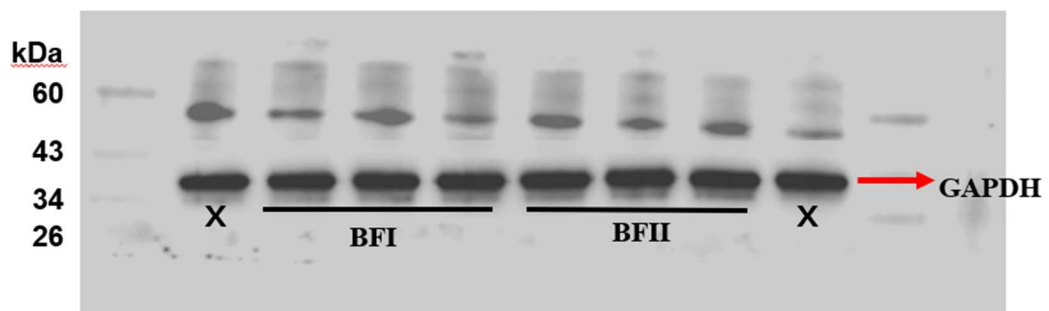

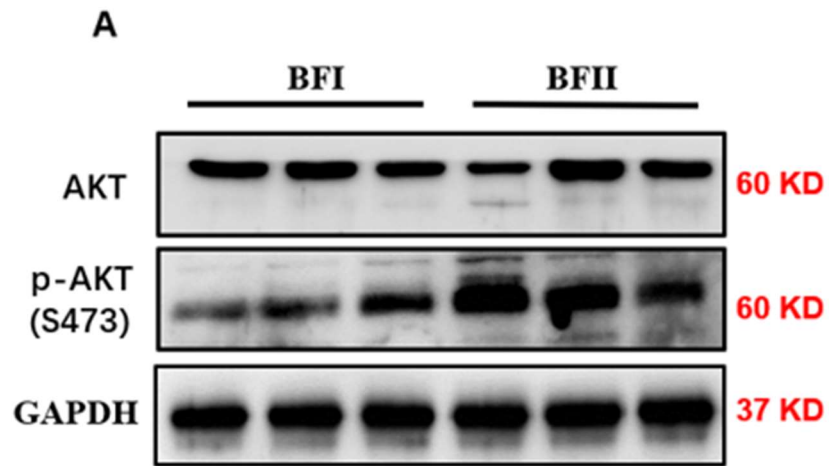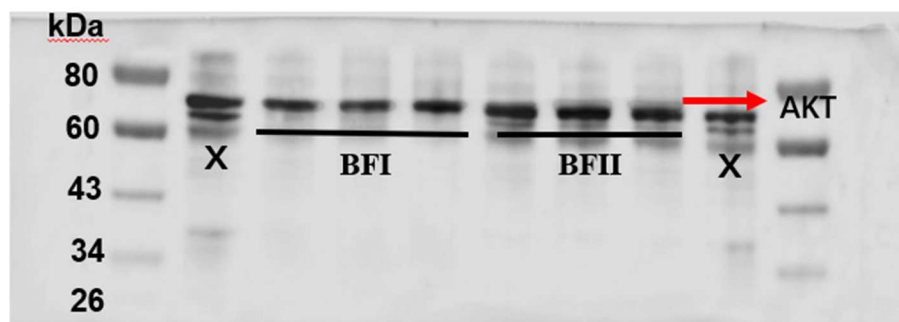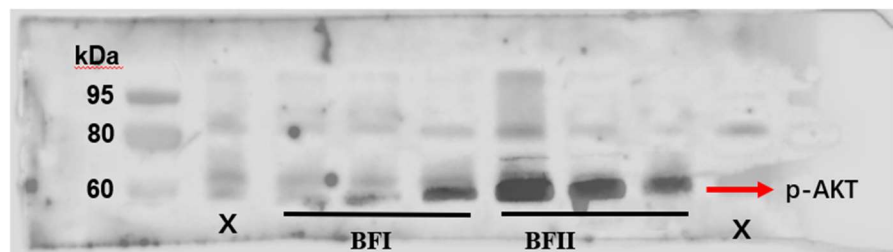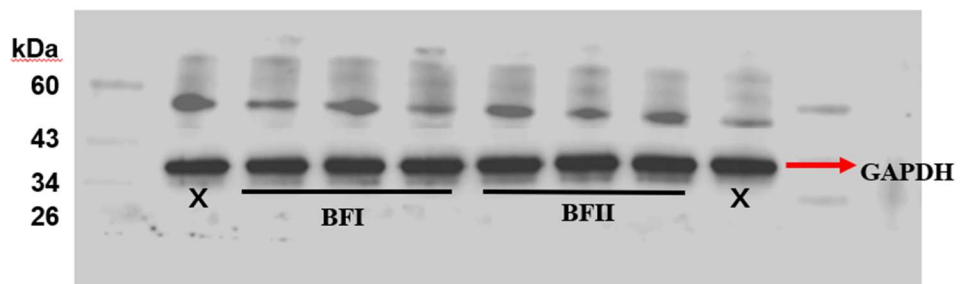

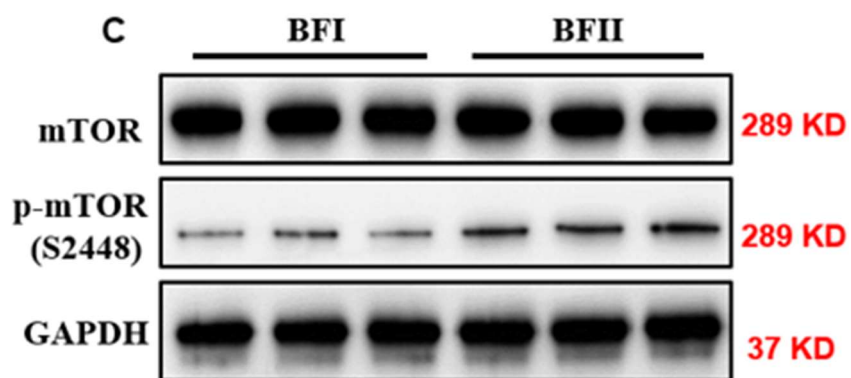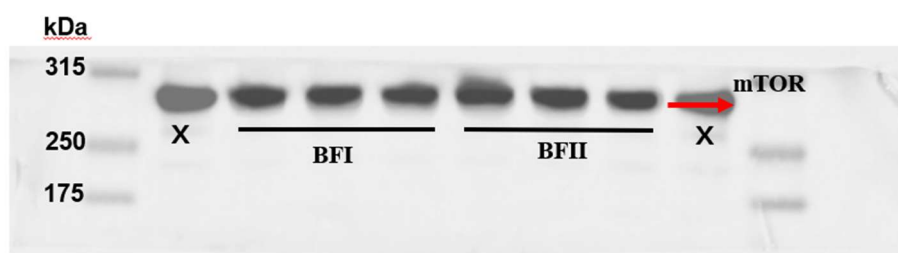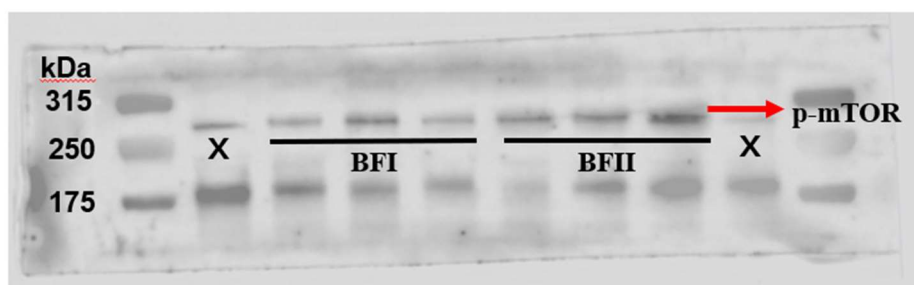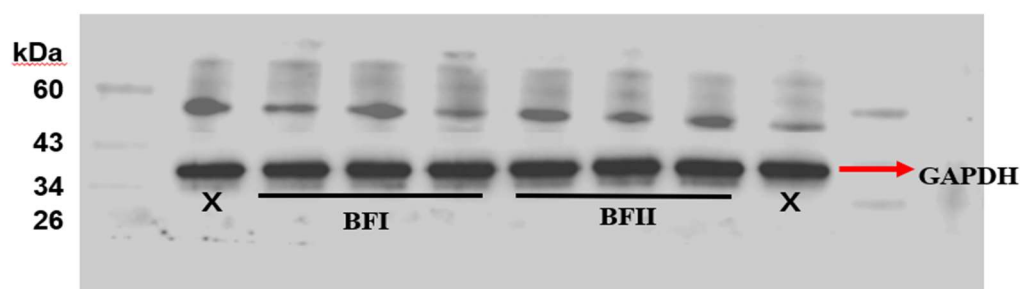

Supplement: Supplementary file 1 [file vetsci-12-00097-s001.zip › Original WB-vetsci-3337115.pdf]
